# Impact of COVID-19 Measures on Short-Term Electricity Consumption in the Most Affected EU Countries and USA States

**DOI:** 10.1016/j.isci.2020.101639

**Published:** 2020-10-05

**Authors:** Javier López Prol, Sungmin O

**Affiliations:** 1 Erwin Schrödinger Fellow at the German Institute for Economic Research (DIW Berlin) and Wegener Center for Climate and Global Change - University of Graz, Graz, Austria; 2 Max Planck Institute for Biogeochemistry, Jena, Germany

**Keywords:** Energy Policy, Energy Engineering, Energy Resources, Energy Management

## Abstract

As COVID-19 spreads worldwide, governments have been implementing a wide range of measures to contain it, from movement restrictions to economy-wide shutdowns. Understanding their impacts is essential to support better policies for countries still experiencing outbreaks or in case of emergence of subsequent pandemic waves. Here we show that the cumulative decline in electricity consumption within the 5 months following the stay-home orders ranges between 3% and 12% in the most affected EU countries and USA states, except Florida, which shows no significant impact. Italy, France, Spain, California, Austria, and New York have recovered baseline consumption by the end of July, whereas Great Britain and Germany remain below baseline levels. We also show that the relationship between measures stringency and daily decline in electricity consumption is nonlinear. These results illustrate the severity of the crisis across countries and can support further research on the effect of specific measures.

## Highlights

• COVID-19 measures decreased electricity consumption by 3%–12% in 5 months
• Most countries/states have recovered baseline levels by the end of July
• Measures stringency and consumption decline are directly and non-linearly linked
• Counterfactual daily electricity consumption is provided

## Graphical Abstract

**Figure:**
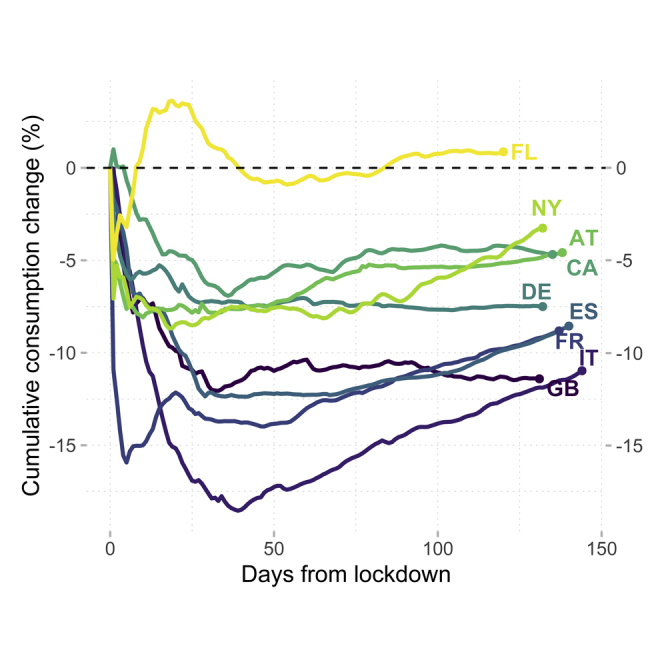

## Introduction

From social distancing guidelines to strict lockdowns and paralyzation of non-essential economic activity, governments worldwide have taken a wide range of measures to halt the spread of the COVID-19 pandemic (Hale et al., 2020a). These measures have multiple implications. Global CO_2_ emissions decreased by 17% during forced confinements (Le Quéré et al. 2020), and global GDP is expected to decline by 3% in 2020 as a result of the pandemic (IMF, 2020a). The economic contraction in advanced countries will double the world average, and it could be as high as 9% in the most affected countries, such as Italy. As an illustration, the strongest impact of the 2003 SARS coronavirus epidemic was in China and Hong Kong with GDP losses of 1.1% and 2.6%, respectively, and a global GDP decline of less than 0.1% (Lee and McKibbin, 2004). Given the unprecedented nature of this crisis, governments are uncertain about the economic impacts of the implemented measures (IMF, 2020b). The unfolding outbreaks in other countries (World Health Organization, 2020) beyond the ones studied here and the potential emergence of subsequent pandemic waves (Kluge, 2020) reveal the urgency to improve our knowledge about the potential impacts of the containment measures.

Given the relationship between electricity consumption and GDP (Hirsh and Koomey, 2015) and the real-time availability of electricity consumption data, analyzing the evolution of electricity consumption may serve as an early warning indicator to assess the impact of containment measures on overall economic activity. Early attempts to track the evolution of electricity consumption during the pandemic have been made by the Bruegel institute (McWilliams and Zachmann, 2020), which provides information on temperature-adjusted peak-hour electricity consumption in European countries compared with the previous year. There are also studies assessing early impacts in the United States (Agdas and Barooah, 2020) and Europe (Cicala, 2020). The International Energy Agency provides a broader analysis of the impact of COVID-19 on the energy sector (IEA, 2020), and Gillingham et al., 2020 estimate the short- and long-term impacts on energy and the environment in the United States. Several media outlets have also provided information on the fall of electricity consumption in different countries compared with previous years' weekly or monthly averages (Morison, 2020; Bui and Wolfers, 2020). Most recently, Ruan et al., (2020) estimated the impact of COVID-19 on electricity consumption in the United States. Our studies concur in providing a counterfactual baseline estimation but differ in the input data, estimation method, and spatial coverage and resolution.

Given that electricity consumption is determined by many factors such as temperature, trends, seasonal cycles, calendar effects, and short-term dynamics (Fan and Hyndman, 2012), ignoring such factors would bias the results (See Figure S14). Additionally, the resulting data and a reproducible method should be publicly available to support further research. For these reasons, we forecast baseline daily electricity consumption in a counterfactual “business as usual” scenario in which COVID-19 did not take place and then compare the forecast with actual electricity consumption in the nine most impacted European countries and USA states. We estimate daily electricity consumption with country-specific dynamic harmonic regressions with Fourier terms for complex seasonality, quadratic temperature, and calendar effects (Hyndman and Athanasopoulos, 2018). This allows us to build a reliable counterfactual baseline scenario with test accuracy ranging between 2.7% and 4.6% mean average percentage error (see Tables S1–S9), which is within the range of the 1-day ahead forecast accuracy benchmark set in the literature (Jun and Ergün, 2011). We have evaluated the most widely used time series forecast methods and opted for the dynamic harmonic regression as it provides the best accuracy results and lowest spread across countries (see Transparent Methods in the Supplemental Information for details).

Our approach enables a reliable estimation of counterfactual baseline electricity consumption against which to compare actual data. We analyze the decline in electricity consumption in the most affected European countries and USA states and link it with the stringency of the measures taken to contain the pandemic. We find that all the studied countries/states, except Great Britain and Germany, have recovered baseline electricity consumption by the end of July 2020. Furthermore, we reveal the non-linear relationship between the stringency of the containment measures and the decline in electricity consumption. This could entail that moderate measures may have only a small effect on electricity consumption and thus economic activity. Moreover, data and code used for our analysis are publicly available so the estimation can be extended to other countries/states and support further research on the effect of specific measures, the evolution of economic activity or the relationship with other high-frequency indicators.

## Results

### Electricity Consumption Decline

**Figure 1:**
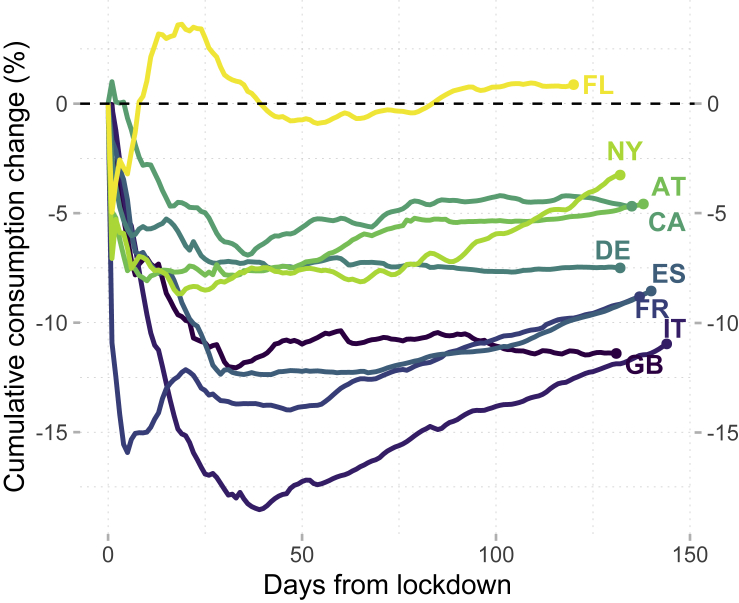
Impact Curve Flattens in Most Countries in About a Month After the Start of the Lockdown/Stay-Home Orders Lines represent the cumulative change in electricity consumption compared with the forecast baseline levels. Country codes: FL, Florida; NY, New York; AT, Austria; CA, California; DE, Germany; ES, Spain; FR, France; IT, Italy; GB, Great Britain.

Figure 1 shows the cumulative change in electricity consumption since the lockdown/stay-home order in each country/state until the end of July 2020. The severity of both the outbreaks and the lockdown and complementary measures taken by governments to halt the COVID-19 spread differ widely across countries, and therefore the electricity consumption evolution also varies. Most of the studied countries have experienced a negative cumulative impact of between 3% and 12% within the 5 months following the start of the crisis, except Florida, which has not suffered a significant negative impact with respect to the baseline scenario.

**Figure 2:**
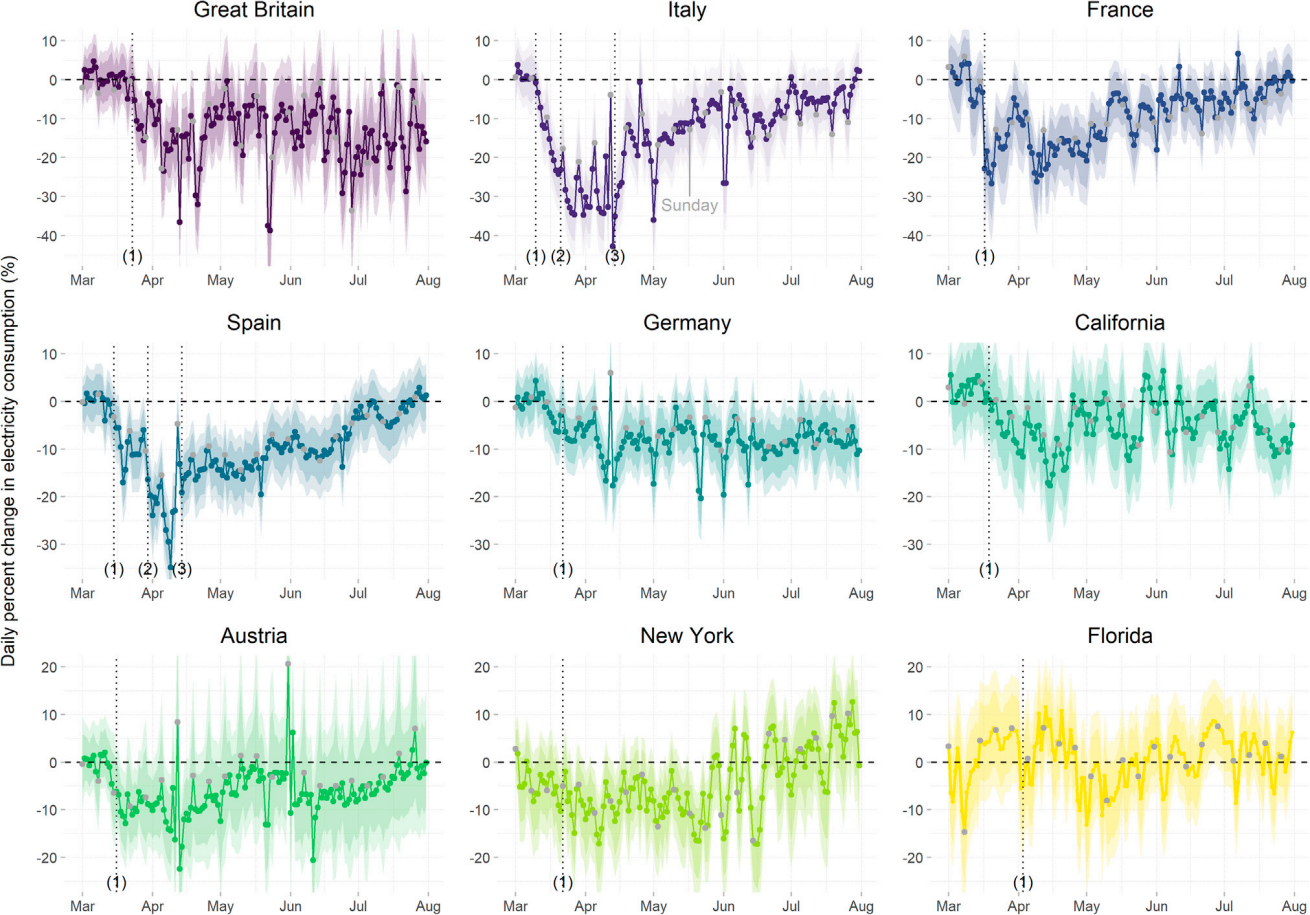
Different Containment Measures Across Countries Led to Different Impacts on electricity Consumption Solid lines show the daily percentage change in electricity consumption. Dark and light shades indicate 80% and 95% prediction intervals, respectively. Sundays are colored gray. Vertical dotted lines indicate the start of (1) lockdown/stay-home orders, (2) non-essential economic activity shutdown, and (3) progressively resuming non-essential economic activity. Note that vertical axis ranges are different for each row. See Supplemental Information for details and Figure S4 for absolute values.

Figure 2 provides greater detail for each particular country/state, presenting the daily percentage change in electricity consumption compared with the expected counterfactual baseline (see Figure S4) for the actual and forecast electricity consumption in absolute terms. Countries are sorted and colored (darker to lighter) according to the cumulative impact during the study period as shown in Figure 1. The dates of the national/state-wide lockdowns/stay-home orders are indicated on each of the panels by vertical dotted lines. Additionally, for Italy and Spain, where there was a shutdown of non-essential economic activity, subsequent vertical dotted lines indicate the date of the beginning of the shutdown and the progressive re-opening of economic activity.

The stringency and scope of these measures differ widely across countries. For instance, Italy issued the first lockdown affecting 50,000 people already on February 21. It was extended to Lombardy and other 14 northern provinces on March 8 and finally to the whole country from March 10. Likewise, measures were implemented at different times and scales in the different German federal states. Other countries, such as France and Spain, implemented the lockdown homogeneously across the country.

Italy and Spain are particularly interesting as three phases are identifiable: (1) a first lockdown phase, (2) a second phase of non-essential economic activity shutdown, and (3) a subsequent progressive re-opening of economic activities. During the non-essential economic activity shutdown, daily electricity consumption declined on average 29% daily in Italy and 21% in Spain compared with the baseline. Electricity consumption started recovering in Italy and Spain with the progressive re-opening of economic activities and reached baseline levels by the end of July.

Great Britain experienced the strongest cumulative decline in electricity consumption of 11.4%. Whereas the initial impact was not as strong as in other countries such as Italy or France, electricity consumption in Great Britain has consistently remained below baseline levels and shows no sign of recovery. Conversely, France experienced an instant 20% decline with the beginning of the lockdown but has already recovered baseline electricity consumption. The European countries that experienced a stronger decline in the first weeks (Italy, France, and Spain) have recovered faster than those with lower initial declines (Germany and Great Britain). These results could suggest that stronger initial action reduces the duration of the shock. Austria lies between these two types of impacts, with an initial impact of −10% that recovers in 2 months, followed by a slight relapse in June that recovers again in July.

Generally, the impact of COVID-19 measures on electricity consumption has been lower and the recovery faster in the studied USA states than in the European countries. Variability in the estimates is also higher in the USA states, perhaps owing to the presence of confounding factors such as the protests at the end of May–beginning of June. Florida did not even experience a net negative impact.

### Measures Stringency

The depth of the consumption decline is directly related to the stringency of the containment measures. The stringency index, estimated by the Coronavirus response tracker (Hale et al., 2020a, 2020b), is composed of nine policy response indicators ranging from information campaigns to movement restrictions (see Supplemental Information for full list). Each of these individual indicators is measured in an ordinal scale depending on stringency (e.g., whether a measure is only a recommendation or an obligation) and scope (i.e., whether the measure is general or targeted to a specific group or region). The stringency index aggregates each of these rescaled individual indicators to reach a score between 0 and 100 (see Figure S3).

**Figure 3:**
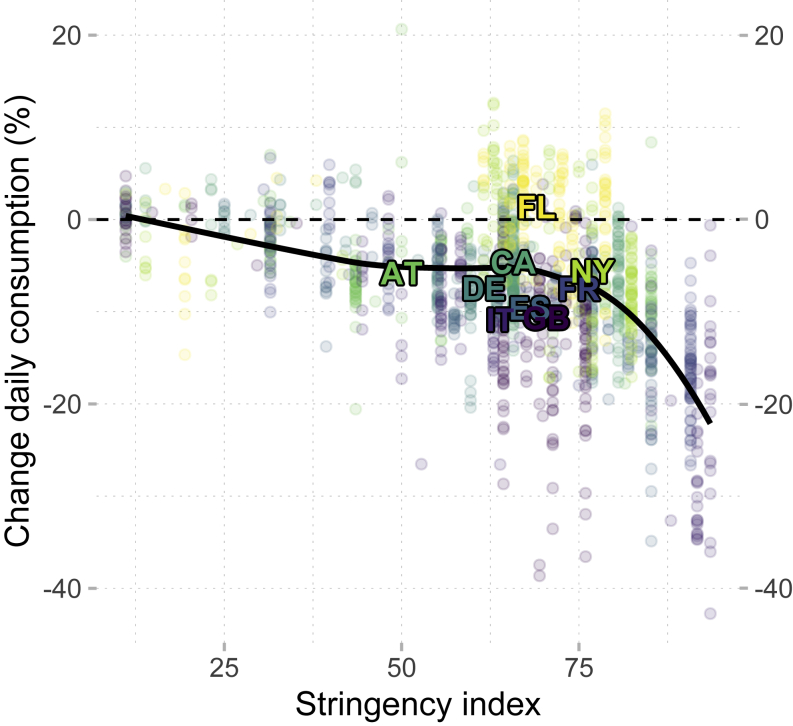
The Stronger the Measures Stringency, the Greater the Consumption Decline Each dot represents the daily electricity consumption change and stringency index for each country. The country codes indicate the median values for each country. The black line represents the relationship between electricity consumption and stringency.

Figure 3 shows the relationship between the daily drop in electricity consumption (Figure 2) and the stringency of the COVID-19 measures. The dots represent the drop in electricity consumption and the stringency index for each day and country/state during the study period, and the solid black line represents the relationship between both variables. The country codes represent the median value for each of the countries during this period, revealing that the stronger the stringency, the higher the electricity consumption decline. The non-linear shape of this relationship suggests that moderate measures may have a small impact on electricity consumption and thus economic activity. Although this is only a high-level illustration, as more data are generated on both the evolution of the stringency across countries and the evolution of electricity demand, these two measures will reveal the impact of the different COVID-19 measures on electricity consumption and therefore on economic activity.

## Discussion

We estimate the impact of COVID-19 containment measures on electricity consumption by comparing the counterfactual baseline “business as usual” consumption forecast with actual data. We have identified large differences across countries/states, from cumulative contraction beyond −10% in Great Britain and Italy to no net negative impact in Florida. Italy, France, Spain, California, Austria, and New York have recovered baseline consumption levels within 5 months since the first outbreak, whereas Great Britain and Germany remain below baseline levels. If this situation persists after all containment measures are lifted, this could reveal either a structural impact on economic activity or a structural change in the relationship between GDP and electricity consumption.

There are multiple mechanisms through which this short-term shock could have structural economic effects. From the demand side, the immediate effects of the social distancing measures may disrupt businesses that rely on personal interaction (Koren and Peto, 2020). From the supply side, halting non-essential activities may have propagation effects across the supply chain to other regions and sectors (Inoue and Todo, 2020). An increase in uncertainty, such as the one caused by this pandemic (Baker et al., 2020), affects both demand by lower consumer spending and supply by lower investment and capital formation. The labor market could also be a transmission mechanism as the crisis affects mostly workers that need a long time to be employable again (Gregory et al., 2020). Finally, a financial mechanism through which higher private and public indebtedness slows down potential long-term growth could also come into play (Dar and Amirkhalkhali, 2014; Cecchetti and Zampolli, 2011).

If the economic contraction caused by the COVID-19 measures turns out to be L-shaped for some countries, this would contrast with previous epidemics that have generally caused transient V-shaped shocks (Carlsson-Szlezak et al., 2020), revealing the unprecedented nature of this crisis and the urgent need for further research to understand the implications of the pandemic and the measures taken by governments to contain its spread. The counterfactual baseline electricity consumption data provided here are publicly available (see below repository link) and can thus help in that direction by providing an estimate of the drop in electricity consumption due to the crisis. Furthermore, our results can contribute to estimating the effects of specific policies (Hale et al., 2020a), to assess the relationship with other real-time indicators, such as mobility (Google, 2020) or electronic payments (Aprigliano et al., 2019), or to nowcast economic activity (Buono et al., 2017).

### Limitations of the Study

As this is an evolving situation, these results will need to be updated periodically and could be extended to other countries and regions to obtain more comprehensive conclusions. Likewise, given the heterogeneity found across countries, more detailed studies at a higher resolution will be beneficial to better understand the impact of specific COVID-19 containment measures on particular sectors and economic activities. Other potential extensions relate to the relationship between electricity consumption and other high-frequency indicators to nowcast economic activity.

Our results can be further improved with newly updated data. Although we have used real-time electricity consumption data, these data are updated several times after the first release with increased quality. For this reason, later studies with newer versions of these data may provide results with a lower error. Additionally, it is hard to evaluate stringency data quality, as stringency has an inherently qualitative aspect. Likewise, actual enforcement might not be correlated with stringency and may vary across countries, which may increase the noise in our results.

Finally, in terms of methods, we have selected a forecast model that can make a compromise between accuracy and generalizability. More accurate modeling, including microdata or more detailed specifications, are likely possible but less able to make comparisons across countries.

### Resource Availability

#### Lead Contact

Javier López Prol: javier.lopez-prol@uni-graz.at.

#### Materials Availability

This study did not generate new unique reagents.

#### Data and Code Availability

Data and code are available on https://github.com/jlprol/covid. The document “replication.Rmd” provides the instructions and basic code for the replication of the main results. See https://jlprol.shinyapps.io/covid/ for interactive figures and easy data download. For further research, please use and cite the following dataset on Mendeley Data: http://dx.doi.org/10.17632/ffryvnskb9.1.

## Methods

All methods can be found in the accompanying Transparent Methods supplemental file.

## Supplemental Information

Document S1. Transparent Methods, Figures S1–S14, and Tables S1–S19

## Footnotes

Supplemental Information can be found online at https://doi.org/10.1016/j.isci.2020.101639.
