# Supplementary material for: Impact of COVID-19 Measures on Short-Term Electricity Consumption in the Most Affected EU Countries and USA States
Source: iScience. 2020 Oct 5;23(10):101639. doi: 10.1016/j.isci.2020.101639 (PMC7534594; doi:10.1016/j.isci.2020.101639)
Supplement: Document S1. Transparent Methods, Figures S1–S14, and Tables S1–S19 [file mmc1.pdf]

iScience, Volume *23*

Supplemental Information

Impact of COVID-19 Measures on Short-Term Electricity Consumption in the Most Affected EU Countries and USA States

Javier López Prol and Sungmin O

1 Transparent methods

1.1 Accuracy and method selection

We have compared forecast business as usual daily electricity consumption with actual consumption data from March to July 2020 to estimate the effect of the COVID-19 measures on electricity consumption. Before deciding to use dynamic harmonic regression to estimate the baseline, we tried four different methods:

(i) Seasonal and trend decomposition using loess forecasting (STLF) is a univariate method that consists in decomposing the time series into three structural components: a trend capturing the long-term evolution of the time series, a seasonal pattern of constant frequency and a remaining error capturing the randomness of the data. This is a relatively simple model that works well when there is no more information available than the time series and there are clear seasonal and trend patterns in the data, but fails to capture complex dynamics as those present in our long-term daily time series.

(ii) Trigonometric seasonality with Box-Cox transformation, ARMA errors, trend and seasonal components (TBATS). This model is more complex than the previous, as it allows for autoregressive and moving average components (ARMA) to capture short-term dynamics, Box-Cox transformation for variance stabilisation and Fourier terms for complex seasonality, in addition to the seasonal and trend components common to the STLF.

(iii) Neural network autoregression $NNAR(p, P, k)_m$ where p is the order of the time series lags that are included as predictors of the network and k is the number of nodes that form the network. P is the order of the seasonal lags with frequency m. We run a feed-forward network with one hidden layer where all the parameters are automatically learned from the data. Seasonality is set to 365 (yearly) and weekly seasonality is modelled with a weekday categorical variable. Two more predictors are included: maximum temperature and a holiday dummy. Neural networks are very flexible and perform well when there are many variables which relationship with the outcome is unknown ex-ante.

(iv) $ARIMA(p, d, q)$ dynamic harmonic regression, where p indicates the order of the autoregressive terms, d is the order of integration and q denotes the moving average component, with Fourier terms for complex seasonality. The dynamic regression performs well when the relationship between predictors and outcomes is known. As shown in Figure S2, we include maximum temperature in quadratic form as the main driver of electricity demand. We also include a holiday dummy to control for moving calendar effects such as Easter. Complex seasonality (weekly and annual) is captured by Fourier terms of order (j, k) respectively. Fourier terms capture seasonality through (j, k) pairs of sines and cosines. Finally, short-term dynamics are captured by the ARMA components.

To compare the accuracy of these methods, we split the data into training set (years 2015–2018 both included) and test set (2019) and evaluate their accuracy with five different metrics. TBATS perform best for Austria but shows high accuracy differentials across countries, which makes it unsuitable for our purposes. NNAR performs best in countries that have the most irregular consumption patterns but is outperformed by the dynamic harmonic regression in most countries. Finally, dynamic harmonic regression performs best in most countries and shows the lowest spread across accuracy estimates, such that the differences with NNAR accuracy is low when the latter performs better, and the results are comparable across countries (see Tables S1-9 for detailed accuracy results). Finally, the selected model is trained with all the data until February 2020, and the forecast is predicted from March using actual temperature data. We use maximum daily temperature data as it shows better prediction accuracy than the average. Temperature data is collected from Automated Surface Observing System (ASOS) stations, which are spatially distributed throughout the countries, and take the median of the maximum temperature across all available stations in each country/state.

1.2 ARIMA dynamic harmonic regression

Equation (1) indicates the regression specification

$$\begin{aligned} y_t = \alpha + \beta_1 T_t + \beta_2 T_t^2 + \beta_3 H_t + \\ \sum_{j=1}^{J} \big(\gamma_{1,j} s_j(t) + \gamma_{2,j} c_j(t)\big) + \\ \sum_{k=1}^{K} \big(\gamma_{3,k} s_k(t) + \gamma_{4,k} c_k(t)\big) + \\ \sum_{p=1}^{P} \phi y_{t-p} + \sum_{q=1}^{Q} \theta \varepsilon_{t-q} + \epsilon_t \quad (1) \end{aligned}$$

where electricity consumption in day t y_t is modelled as a function of a constant α, temperature in a quadratic form ($\beta_1 T_t + \beta_2 T_t^2$) and a dummy variable of state-specific holidays H_t. Complex seasonality is captured by Fourier terms of the form:

$$s_j(t) = \sin(\frac{2\pi j t}{7}) \quad ; \quad c_j(t) = \cos(\frac{2\pi j t}{7})$$
$$s_k(t) = \sin(\frac{2\pi k t}{365.25}) \quad ; \quad c_k(t) = \cos(\frac{2\pi k t}{365.25})$$

where 7 and 365.25 denote the weekly and annual seasonal levels respectively, and (j, k) represent the number of sine/cosine elements for each of the seasonal levels. The last two elements of equation (1) represent the $ARMA(p, q)$ structure that captures short-term dynamics, allowing the error

term of the model to approach as much as possible a normally distributed white noise. Since all time series are integrated of order one, the model is run in first differences and the constant is thus removed. We tried including economic variables such as GDP and unemployment as predictors. However, since they did not improve prediction accuracy (partially due to their lower temporal resolution than our daily prediction), we exclude them from the final specification. Although such economic variables are relevant for long-term forecasts, they do not significantly influence short-term estimations (Jun and Ergün 2011).

The data analysis process can be summarised in the following steps:

1. The time series are transformed following Cox-Box (Box and Cox 1964) to stabilise the variance.
2. The time series are tested for stationarity and differenced if necessary.
3. The optimal $ARMA(p, q)$ structure and Fourier(j, k) order is automatically determined by the Hyndman and Khandakar algorithm (Hyndman and Khandakar 2008) to minimise the corrected Akaike information criteria (AICc).
4. Residuals are studied for signs of remaining signals and the ARMA and Fourier parameters are manually fine-tuned to achieve optimal results according to the following criteria: having the simplest possible model with the lowest possible AICc that shows the closest possible residuals to a normally distributed white noise.
5. Forecast the baseline electricity consumption from March to July 2020 and compare it with the actual values. The point forecast is back-transformed, such that it represents the median, rather than the mean of the forecast distribution. All results are provided with 80% and 95% prediction intervals.

Section 3 provides the results of the process described above: accuracy (3.1), model parameters of points 1-3 (3.2), forecast compared with actual consumption (3.3), regression results (3.4) and their respective residual diagnostics (3.5).

2 Data

We use three different types of data that we describe below in more detail: (i) Electricity consumption (defined as actual load excluding self-consumption) data acquired from the Energy Information Administration of the USA (https://www.eia.gov/) and ENTSO-E (https://transparency.entsoe.eu/) between January (July for the USA) 2015 and July 2020 both included; (ii) Maximum daily temperature from ASOS provided by Iowa Environmental Mesonet (IEM) (https://mesonet.agron.iastate.edu/ASOS/) and defined as the median of the maximum temperature across all available stations within each country/state (excluding islands); and (iii) Stringency index provided by the Blavatnik School of Government of Oxford University (https://www.bsg.ox.ac.uk/research/research-projects/coronavirus-government-response-tracker).

2.1 Electricity consumption

Electricity consumption has been obtained from the ENTSO-E transparency platform for the European countries since January 2015 and from the USA Energy Information Administration for the American states since July 2015, both until July 2020 included. ENTSO-E data corresponds to the country's actual load defined as the sum of power generated by plants on both TSO/DSO networks minus the balance (export-import) of exchanges on interconnections and minus the power absorbed by energy storage resources. EIA demand data comes from the U.S. Electric System Operating Data (EIA-930). In both cases, the data exclude self-consumed electricity. All the data have been collected in UTC and then transformed to local times. Likewise, the original data are in sub-daily resolution and we have aggregated to daily after transforming to their respective local time. Figure S1 shows the daily electricity consumption data for each country/state.

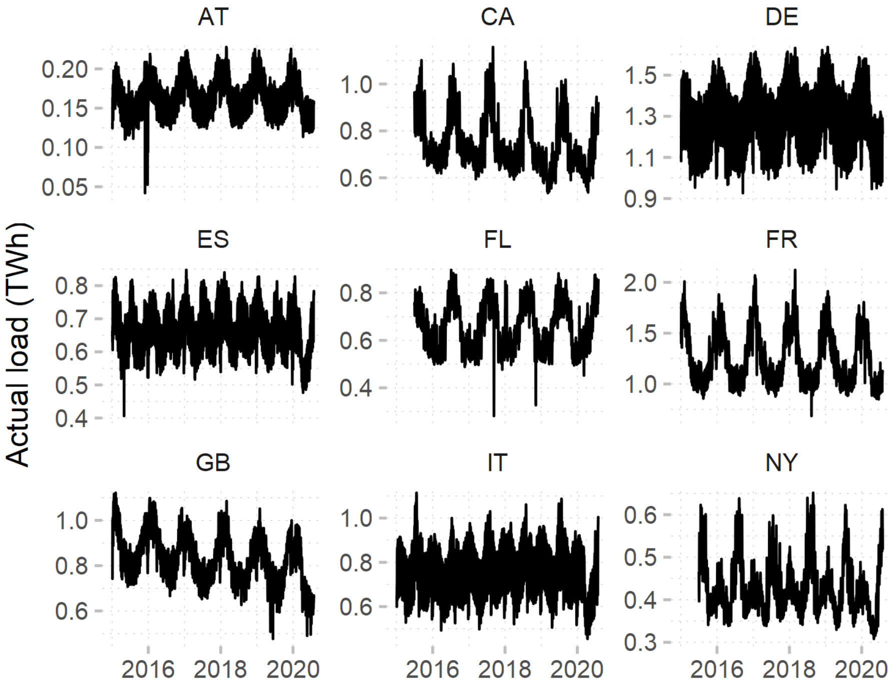

Figure S1. Daily electricity consumption data. Related to Figures 1 and 2.

2.2 Temperature

We tested our models with both mean and maximum daily temperature. Since maximum temperature shows a slightly better predictive performance, we use the maximum rather than the mean. Daily maximum temperature observational data from January 2015 to July 2020 have been obtained from the Automated Surface Observing System provided by Iowa Environmental Mesonet (IEM). ASOS stations are spatially distributed throughout countries and have wide coverage. We first collected daily maximum temperature from all available stations within each country/state excluding islands. We then calculated the median of the maximum temperature across the stations for each day and country/state. Temperature and electricity consumption have a quadratic relationship, as can be seen in Figure S2. For this reason, we control for quadratic temperature in the dynamic harmonic ARIMA regression.

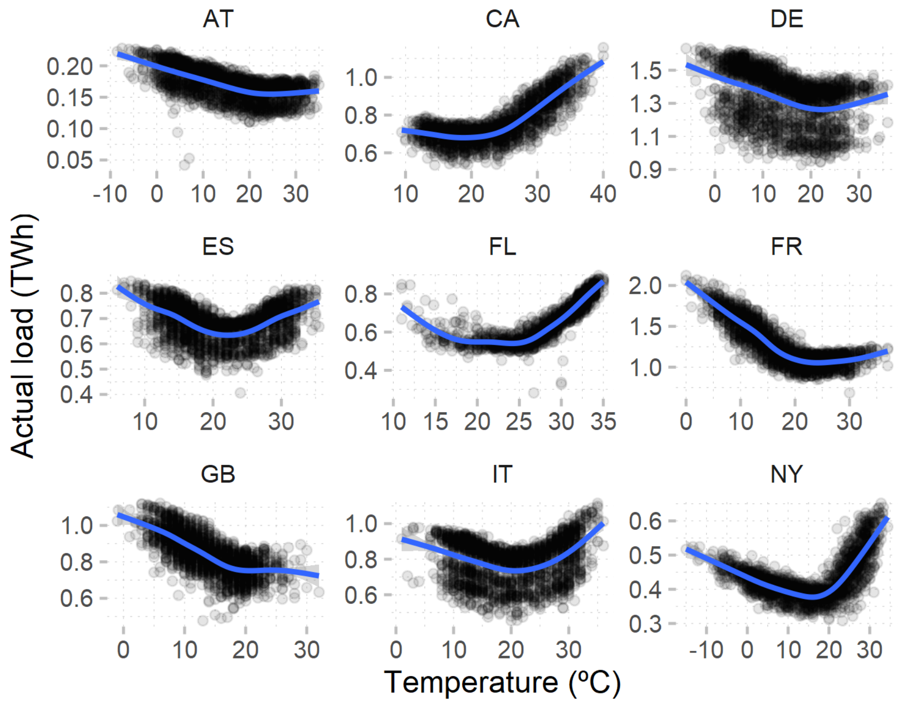

Figure S2. Relationship between daily load and maximum temperature. Related to Figures 1 and 2.

2.3 Stringency index

The stringency index, created by the Blavatnik School of Government of Oxford University and publicly available on the Coronavirus government response tracking website, is composed of nine policy response indicators:

1. School closing
2. Workplace closing
3. Cancel public events
4. Restrictions on gathering size
5. Close public transport
6. Stay at home requirements
7. Restrictions on internal movement
8. International travel controls
9. Public info campaigns

Each of these individual indicators are measured in an ordinal scale depending on stringency (e.g. whether a measure is only a recommendation or an obligation) and scope (i.e. whether the measure is general or targeted to a specific group or region). The stringency index aggregates each of these rescaled individual indicators to reach a score between 0 and 100. Figure S3 shows the evolution of the stringency index for each country/state.

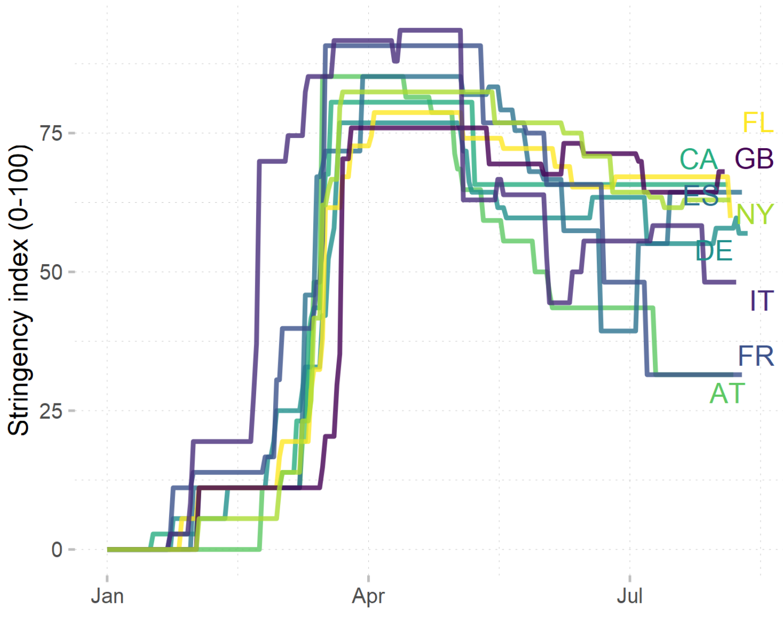

Figure S3. Stringency index. Related to Figure 3.

3 Intermediate results

3.1 Accuracy comparison between different methods.

To test the accuracy of the different methods to forecast daily electricity demand we split the data into training (years 2015-2018) and test (year 2019) sets and evaluate the test set forecast with the actual load data. We present five different accuracy indicators to provide a comprehensive overview of the error of each model and country. Each indicator measures bias and precision differently. The mean error shows the bias of the estimation. The mean absolute error measures precision, but since errors are considered in absolute terms, it does not capture bias. Similarly, the root mean squared error indicates precision penalizing large errors and ignoring its sigh by squaring them. These three indicators are scale-dependent. Both the mean percentage error and the mean absolute percentage error are on the contrary expressed in percent terms, so they are more suitable for comparisons across different consumption levels. Tables S1-9 present the accuracy results for each country and method:

- Accuracy indicators:
  - ME: mean error.
  - RMSE: root mean squared error.
  - MAE: mean absolute error.
  - MPE: mean percentage error.
  - MAPE: mean absolute percentage error.
- Methods
  - STLF: seasonal and trend decomposition using loess forecasting.
  - TBATS: trigonometric seasonality with Box-Cox transformation, ARMA errors, trend and seasonal components.
  - NNAR: neural network autocorrelation.
  - ARIMA: integrated dynamic harmonic regression with Fourier terms for seasonality and ARMA errors.

Table S1. Accuracy indicators Austria. Related to Figures 1 and 2.

	ARIMA	NNAR	TBATS	STLF
ME	-0.01	0.00	0.00	0.00
RMSE	0.01	0.01	0.01	0.02
MAE	0.01	0.01	0.01	0.02
MPE	-3.45	-0.16	-1.61	-2.54
MAPE	4.60	2.97	3.94	11.54

Table S2. Accuracy indicators California. Related to Figures 1 and 2.

	ARIMA	NNAR	TBATS	STLF
ME	0.02	-0.04	-0.03	-0.05
RMSE	0.04	0.06	0.06	0.09
MAE	0.03	0.05	0.05	0.07
MPE	2.56	-5.77	-4.14	-7.72
MAPE	4.64	6.75	6.67	9.70

Table S3. Accuracy indicators Germany. Related to Figures 1 and 2.

	ARIMA	NNAR	TBATS	STLF
ME	-0.01	-0.02	-0.01	-0.02
RMSE	0.06	0.06	0.08	0.17
MAE	0.04	0.04	0.05	0.15
MPE	-1.33	-1.35	-1.01	-3.06
MAPE	2.92	3.06	4.11	11.66

Table S4. Accuracy indicators Spain. Related to Figures 1 and 2.

	ARIMA	NNAR	TBATS	STLF
ME	-0.01	-0.02	0.04	0.00
RMSE	0.02	0.08	0.06	0.07
MAE	0.02	0.06	0.05	0.06
MPE	-1.26	-4.17	5.87	-1.10
MAPE	2.65	9.37	7.25	8.39

Table S5. Accuracy indicators Florida. Related to Figures 1 and 2.

	ARIMA	NNAR	TBATS	STLF
ME	-0.01	0.00	-0.07	-0.01
RMSE	0.03	0.03	0.10	0.06
MAE	0.03	0.03	0.08	0.05
MPE	-1.36	-0.83	-10.45	-2.13
MAPE	4.38	3.92	11.94	7.86

Table S6. Accuracy indicators France. Related to Figures 1 and 2.

	ARIMA	NNAR	TBATS	STLF
ME	-0.02	-0.01	0.01	-0.04
RMSE	0.07	0.06	0.10	0.14
MAE	0.06	0.05	0.07	0.11
MPE	-1.50	-0.97	0.06	-4.27
MAPE	4.42	3.79	5.37	8.81

Table S7. Accuracy indicators Great Britain. Related to Figures 1 and 2.

	ARIMA	NNAR	TBATS	STLF
ME	0.01	-0.01	0.02	0.00
RMSE	0.04	0.05	0.05	0.07
MAE	0.03	0.03	0.04	0.05
MPE	0.31	-1.78	2.49	-0.42
MAPE	3.93	4.06	4.85	6.85

Table S8. Accuracy indicators Italy. Related to Figures 1 and 2.

	ARIMA	NNAR	TBATS	STLF
ME	0.00	0.01	0.01	0.00
RMSE	0.04	0.06	0.07	0.12
MAE	0.03	0.04	0.05	0.10
MPE	0.20	0.82	0.06	-2.33
MAPE	3.55	4.82	6.26	13.63

Table S9. Accuracy indicators New York. Related to Figures 1 and 2.

	ARIMA	NNAR	TBATS	STLF
ME	-0.01	-0.01	-0.01	0.00
RMSE	0.03	0.02	0.03	0.04
MAE	0.02	0.02	0.02	0.03
MPE	-1.86	-1.59	-2.19	0.19
MAPE	4.31	3.91	5.62	6.87

3.2 ARIMA parametrisation

Table S10 presents the regression parameters for each country/state.

Table S10. Model parameters. Related to Figures 1 and 2.

Country	Lambda	Fourier.j.k.	ARIMA.p.d.q.
Austria	1.95	(3,9)	(0,1,4)
California	1.12	(3,3)	(4,1,3)
Germany	0.81	(3,11)	(4,1,1)
Spain	-0.07	(3,23)	(3,1,2)
Florida	1.03	(3,3)	(1,1,2)
France	-1.00	(3,19)	(7,1,6)
Great Britain	1.20	(3,3)	(2,1,1)
Italy	0.98	(3,20)	(3,1,1)
New York	-1.00	(3,5)	(3,1,1)

3.3 Actual vs. forecast (baseline) daily electricity consumption

Figure S4 shows the forecast (black line) produced by each of the country-specific dynamic harmonic ARIMA regression with 80% (dark shade) and 95% (light) prediction intervals. The coloured lines represent the actual electricity consumption.

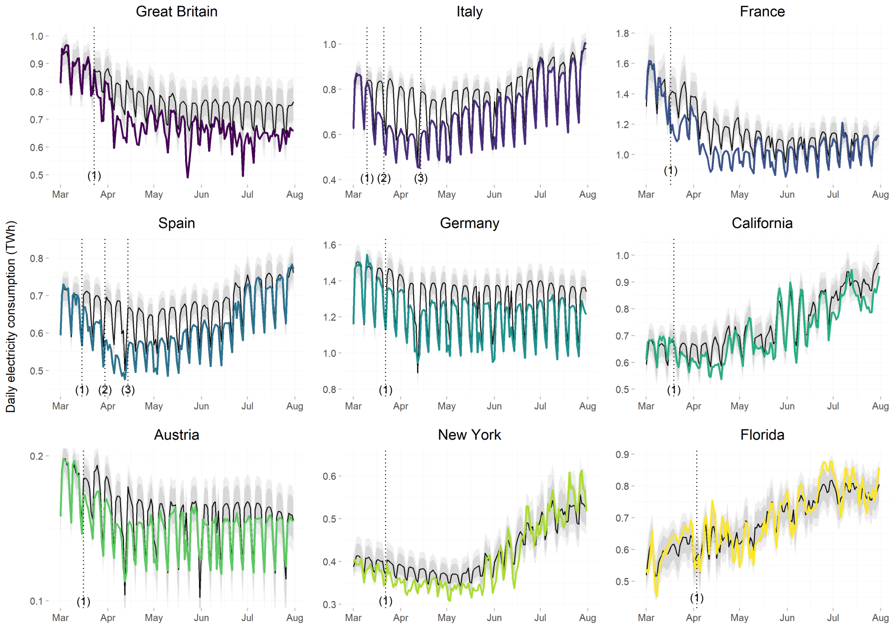

Figure S4. Actual and Forecast daily electricity consumption. Related to Figures 1 and 2.

3.4 Regression results

Tables S11-20 present the regression results for the dynamic harmonic regression of each country. Only the ARMA terms and the external regressors (quadratic temperature and holiday dummy) are included in the tables. Fourier terms have been omitted for simplicity.

Table S11. Austria summary regression results. Related to Figures 1 and 2.

Variable	Coefficient	SE	z-value	p-value
MA1	-0.510	0.279	-1.829	0.067
MA2	-0.213	0.051	-4.178	0.000
MA3	-0.162	0.089	-1.819	0.069
MA4	-0.040	0.160	-0.248	0.804
Temperature	0.000	0.003	-0.127	0.899
Temperature2	0.000	0.000	0.097	0.923
Holiday	-0.003	0.006	-0.538	0.590

Table S12. California summary regression results. Related to Figures 1 and 2.

Variable	Coefficient	SE	z-value	p-value
AR1	0.120	0.087	1.378	0.168
AR2	-0.239	0.154	-1.555	0.120
AR3	0.701	0.126	5.581	0.000
AR4	-0.123	0.046	-2.689	0.007
MA1	-0.132	0.081	-1.622	0.105
MA2	0.031	0.152	0.205	0.837
MA3	-0.870	0.111	-7.867	0.000
Temperature	-0.017	0.000	-111.322	0.000
Temperature2	0.000	0.000	41.394	0.000
Holiday	-0.021	0.002	-13.308	0.000

Table S13. Germany summary regression results. Related to Figures 1 and 2.

Variable	Coefficient	SE	z-value	p-value
AR1	0.528	0.024	22.156	0.000
AR2	-0.071	0.026	-2.668	0.008
AR3	0.085	0.026	3.278	0.001
AR4	-0.050	0.023	-2.136	0.033
MA1	-0.987	0.004	-276.402	0.000
Temperature	-0.005	0.000	-9.491	0.000
Temperature2	0.000	0.000	6.319	0.000
Holiday	-0.166	0.004	-43.530	0.000

Table S14. Spain summary regression results. Related to Figures 1 and 2.

Variable	Coefficient	SE	z-value	p-value
AR1	1.367	0.055	24.954	0.000
AR2	-0.512	0.047	-10.890	0.000
AR3	0.072	0.025	2.847	0.004
MA1	-1.751	0.049	-35.491	0.000
MA2	0.753	0.049	15.275	0.000
Temperature	-0.015	0.000	-45.087	0.000
Temperature2	0.000	0.000	19.427	0.000
Holiday	-0.124	0.003	-37.383	0.000

Table S15. Florida summary regression results. Related to Figures 1 and 2.

Variable	Coefficient	SE	z-value	p-value
AR1	-0.510	0.279	-1.829	0.067
MA1	-0.213	0.051	-4.178	0.000
MA2	-0.162	0.089	-1.819	0.069
Temperature	-0.040	0.160	-0.248	0.804
Temperature2	0.000	0.003	-0.127	0.899
Holiday	0.000	0.000	0.097	0.923

Table S16. France summary regression results. Related to Figures 1 and 2.

Variable	Coefficient	SE	z-value	p-value
AR1	0.416	0.062	6.698	0.000
AR2	-0.532	0.079	-6.698	0.000
AR3	0.085	0.100	0.853	0.394
AR4	-0.068	0.089	-0.766	0.444
AR5	-0.402	0.075	-5.360	0.000
AR6	0.362	0.038	9.584	0.000
AR7	0.240	0.026	9.403	0.000
MA1	-0.878	0.061	-14.434	0.000
MA2	0.557	0.102	5.478	0.000
MA3	-0.343	0.120	-2.865	0.004
MA4	-0.070	0.118	-0.596	0.551
MA5	0.367	0.089	4.101	0.000
MA6	-0.622	0.052	-11.875	0.000
Temperature	-0.016	0.000	-51.697	0.000
Temperature2	0.000	0.000	22.272	0.000
Holiday	-0.062	0.003	-20.543	0.000

Table S17. Great Britain summary regression results. Related to Figures 1 and 2.

Variable	Coefficient	SE	z-value	p-value
AR1	0.605	0.029	20.827	0.000
AR2	0.040	0.028	1.433	0.152
MA1	-0.967	0.017	-55.485	0.000
Temperature	-0.012	0.000	-32.393	0.000
Temperature2	0.000	0.000	13.408	0.000
Holiday	-0.058	0.003	-17.751	0.000

Table S18. Italy summary regression results. Related to Figures 1 and 2.

Variable	Coefficient	SE	z-value	p-value
AR1	0.496	0.025	19.575	0.000
AR2	-0.089	0.027	-3.294	0.001
AR3	-0.074	0.024	-3.050	0.002
MA1	-0.965	0.008	-114.172	0.000
Temperature	-0.016	0.000	-36.334	0.000
Temperature2	0.000	0.000	22.053	0.000
Holiday	-0.107	0.003	-32.977	0.000

Table S19. New York summary regression results. Related to Figures 1 and 2.

Variable	Coefficient	SE	z-value	p-value
AR1	0.894	0.029	31.025	0
AR2	-0.305	0.033	-9.180	0
AR3	0.091	0.025	3.703	0
MA1	-0.990	0.004	-225.978	0
Temperature	-0.015	0.001	-13.980	0
Temperature2	0.001	0.000	14.042	0
Holiday	-0.075	0.008	-8.882	0

3.5 Residuals

Figures S5-13 present the residuals of the dynamic harmonic ARIMA regressions. The consumption data (Figure S1) have some outliers that can be observed in the residuals but do not significantly influence the accuracy of the forecast. All the residuals are close to a normally distributed white noise.

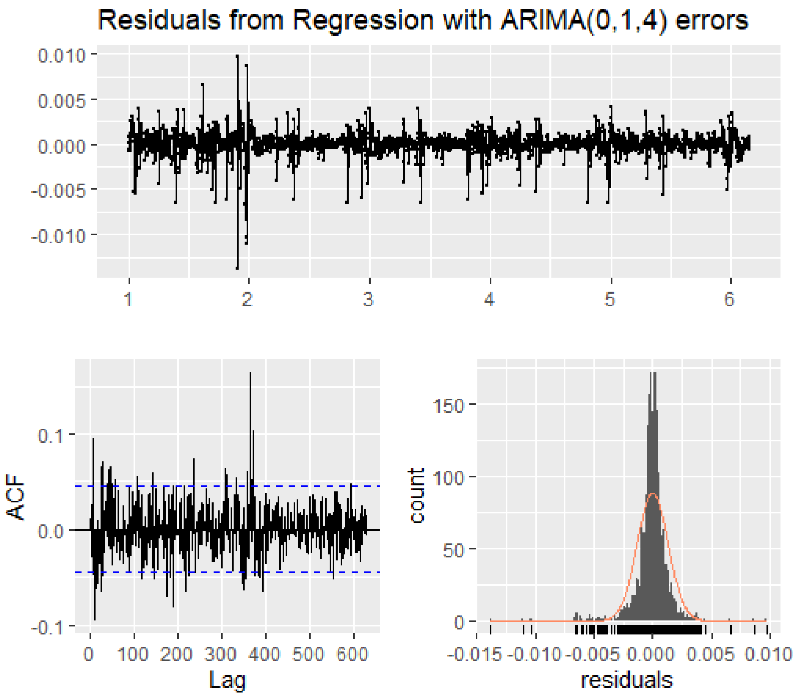

Figure S5. Austria residuals. Related to Figures 1 and 2.

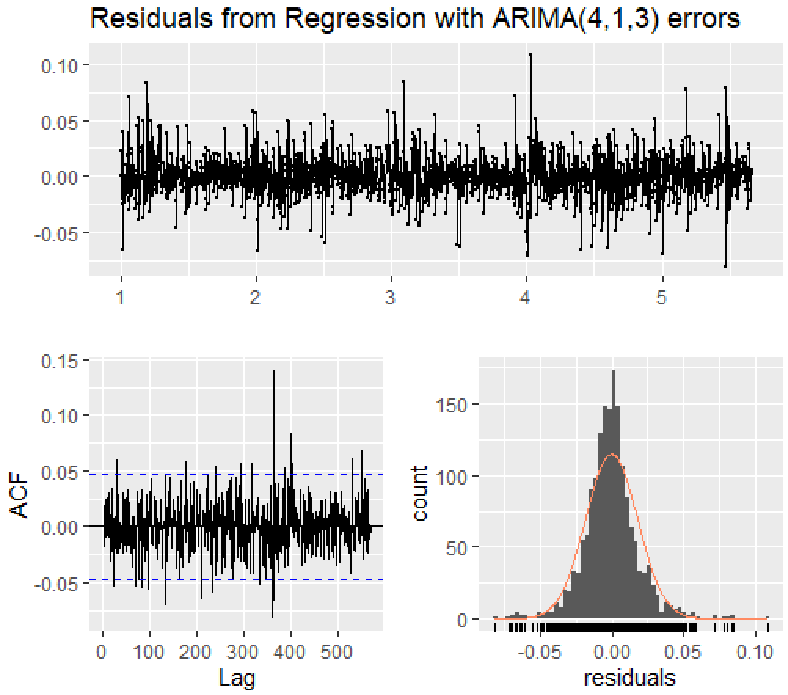

Figure S6. California residuals. Related to Figures 1 and 2.

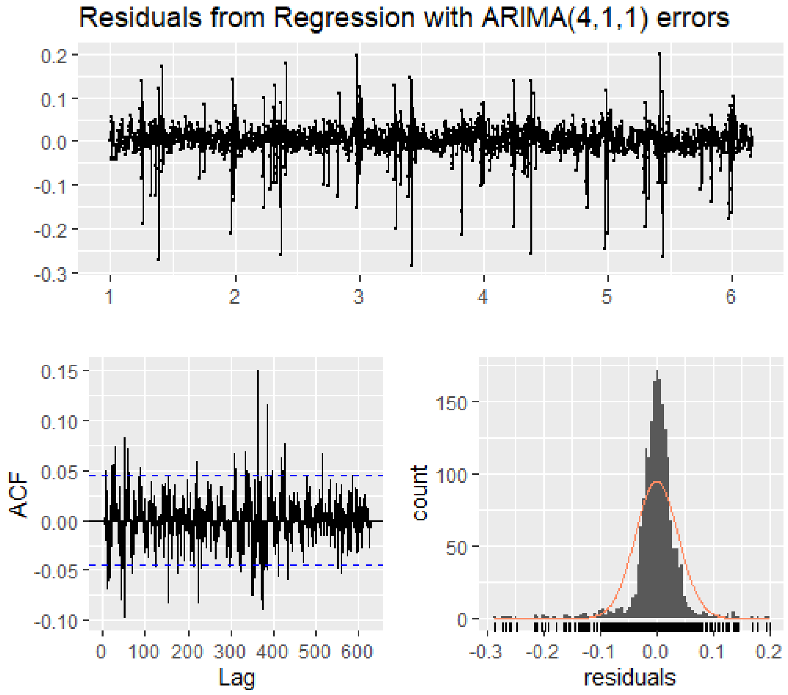

Figure S7. Germany residuals. Related to Figures 1 and 2.

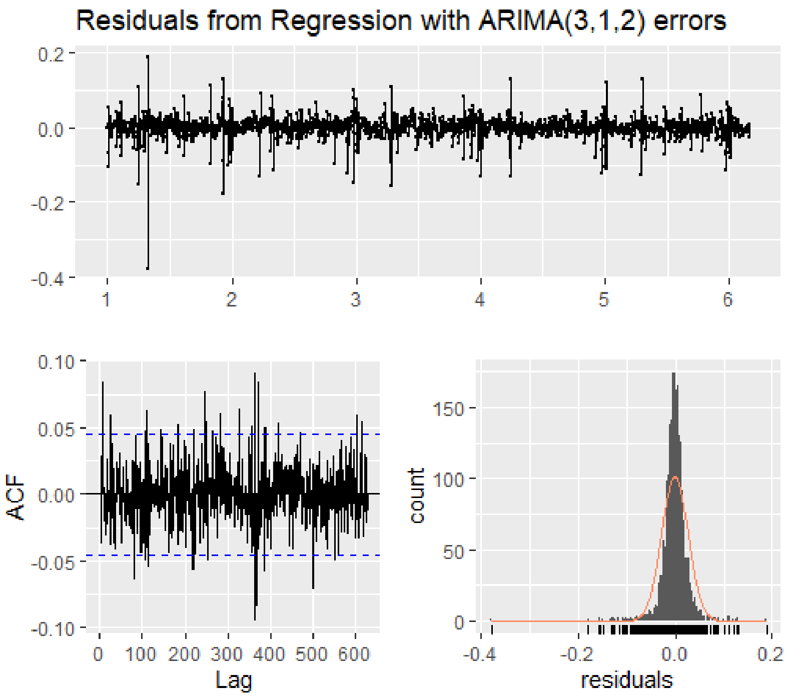

Figure S8. Spain residuals. Related to Figures 1 and 2.

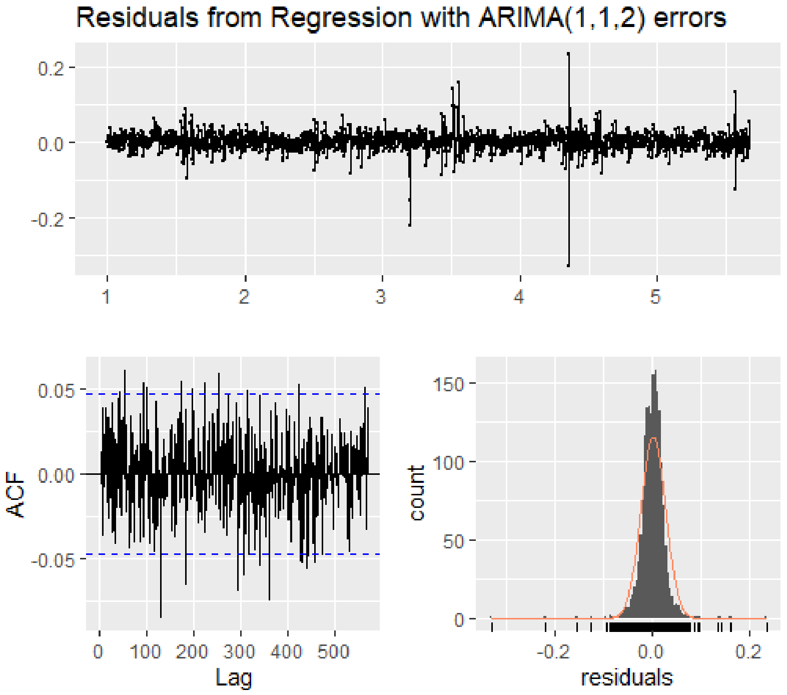

Figure S9. Florida residuals. Related to Figures 1 and 2.

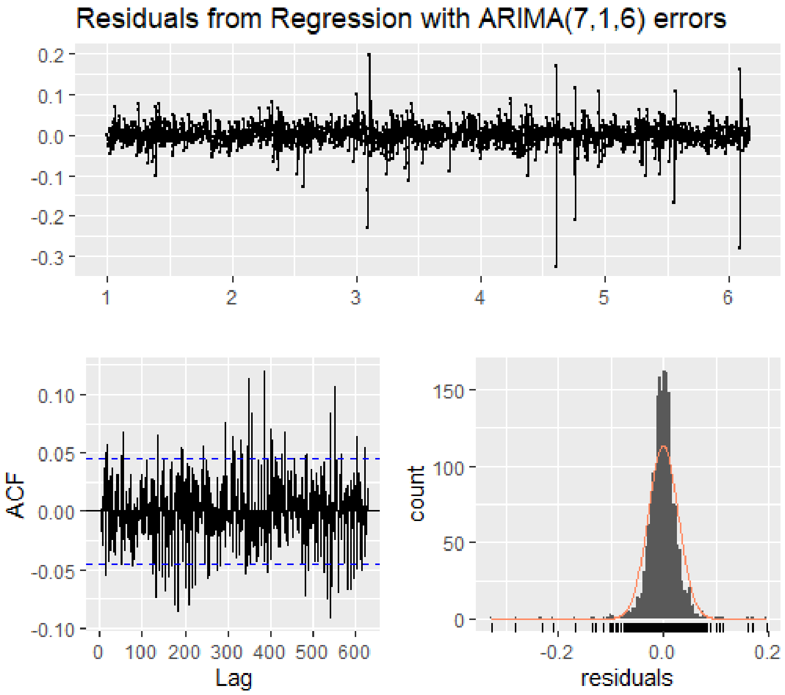

Figure S10. France residuals. Related to Figures 1 and 2.

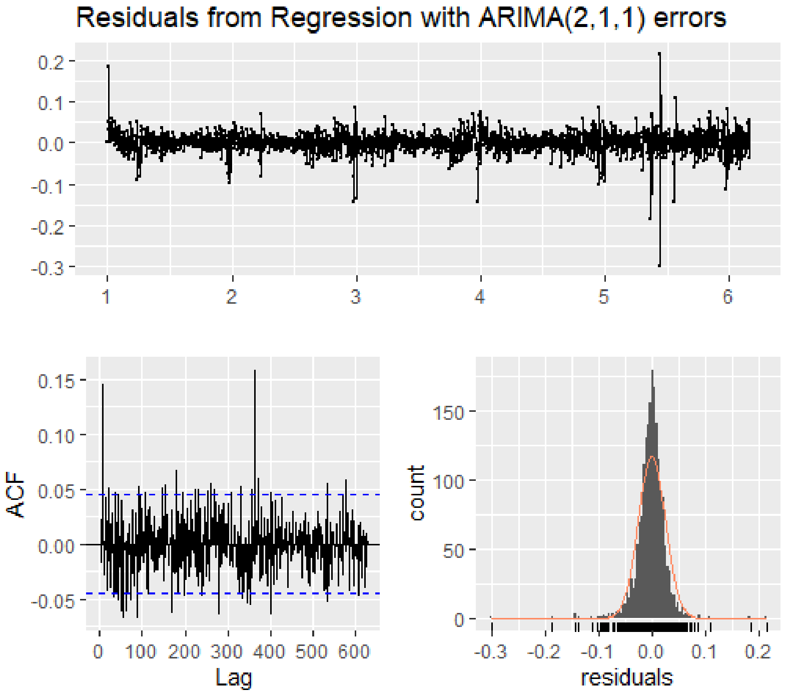

Figure S11. Great Britain residuals. Related to Figures 1 and 2.

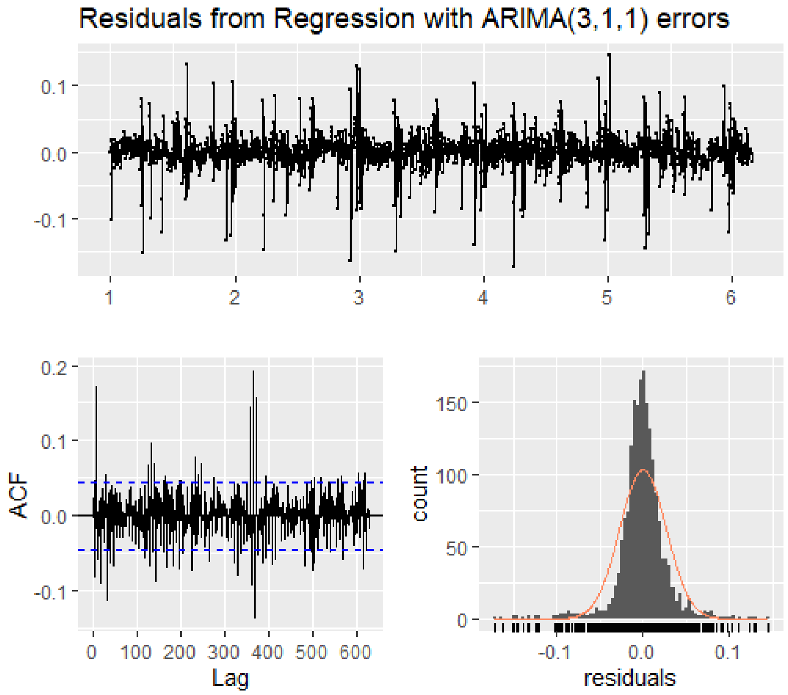

Figure S12. Italy residuals. Related to Figures 1 and 2.

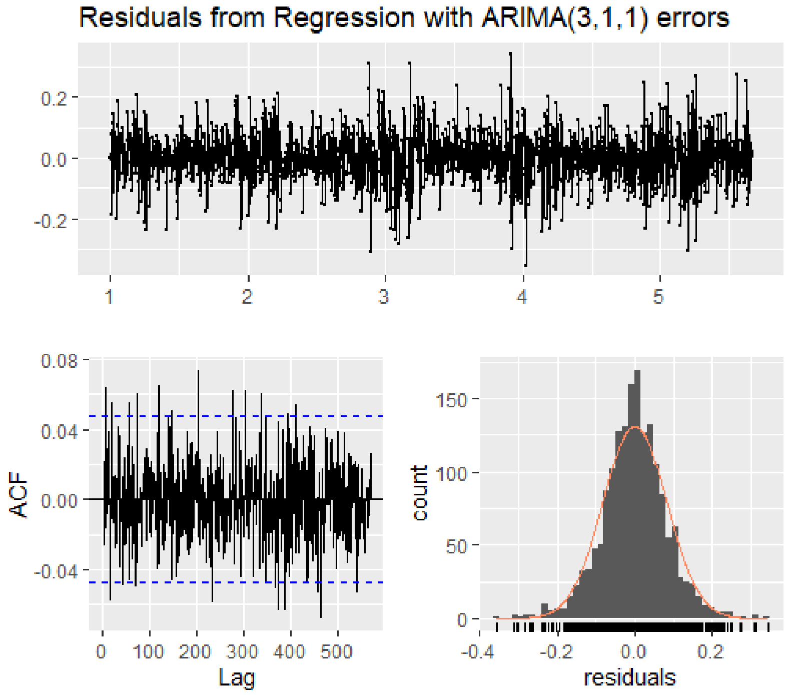

Figure S13. New York residuals. Related to Figures 1 and 2.

3.6 Bias of a naive comparison with last year data

Here we show the bias that would occur if instead of using the dynamic harmonic ARIMA regression to estimate the baseline electricity consumption we had simply taken 2019 electricity consumption. For this purpose, we first calculate the weekly (weeks 12 to 30) change in electricity consumption between 2019 and 2020 (naive estimation) and compare it with the weekly-aggregated results from our method using the dynamic harmonic ARIMA regression (see Figure 2 in the main text). Figure S14 shows the difference between the naive comparison and our main results. Whereas aggregating to weekly already reduces the error by removing weekly seasonality and short-term dynamics, we can see that a naive comparison would overestimate the drop in electricity consumption for most countries/states (except California where it would underestimate it) up to 10 percentage points in some weeks.

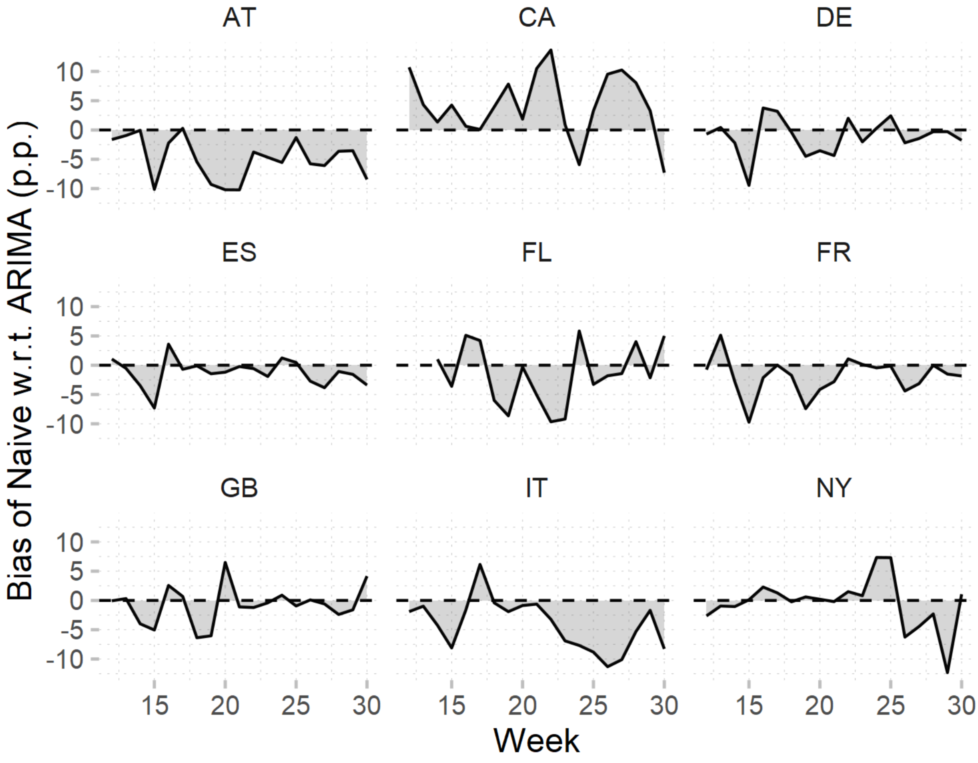

Figure S14. Difference in the change of electricity consumption between the naive estimation and the dynamic harmonic ARIMA regression. Negative means that the simple comparison overestimates the drop in electricity consumption. Related to Figures 1 and 2.

Supplemental references

Box, G E P, and; D R Cox. 1964. "An Analysis of Transformations." *J. R. Stat. Soc. Ser. B* 26 (2): 211–52.

Hyndman, Rob J, and Yeasmin Khandakar. 2008. "Automatic time series forecasting: The forecast package for R." *J. Stat. Softw.* 27 (3): 1–22. https://doi.org/10.18637/jss.v027.i03.

Jun, Jongbyung, and A. Tolga Ergün. 2011. "A more accurate benchmark for daily electricity demand forecasts." *Manag. Res. Rev.* 34 (7): 810–20. https://doi.org/10.1108/01409171111146698.
